# COI Insights into Diversity and Species Delimitation of Immature Stages of Non-Biting Midges (Diptera: Chironomidae)

**DOI:** 10.3390/insects16020174

**Published:** 2025-02-06

**Authors:** Laurynas Stasiukynas, Jekaterina Havelka, Fabio Laurindo da Silva, Maria Fernanda Torres Jimenez, Sigitas Podėnas, Aistė Lekoveckaitė

**Affiliations:** 1 Life Sciences Center, Vilnius University, Saulėtekio av. 7, LT-10257 Vilnius, Lithuania; jekaterina.havelka@gf.vu.lt (J.H.); fernanda.torres@gmc.vu.lt (M.F.T.J.); sigitas.podenas@gamtc.lt (S.P.); aiste.lekoveckaite@gmc.vu.lt (A.L.); 2 Department of Zoology, Institute of Biosciences, University of São Paulo, Rua do Matão, Trav. 14, n.101, São Paulo 05508-090, Brazil; fabiologia@gmail.com

**Keywords:** ASAP, ABGD, GMYC, bPTP, phylogeny, Lithuania

## Abstract

The diversity of non-biting midges (Chironomidae, Diptera) remains an unresolved topic, with estimates of species numbers ranging from 6000 to 15,000 according to various authors. To assess Chironomidae diversity in Lithuania, we evaluate the effectiveness of COI gene-based species delimitation methods for providing rapid diversity estimates. Nevertheless, differences between tree-based and distance-based approaches can result in varying group classifications, which may cause species numbers to be overestimated or underestimated. For our study, we analyzed a dataset of 109 specimens sampled from six Lithuanian streams. By applying multiple methods, such as Assemble Species by Automatic Partitioning (ASAP), Automatic Barcode Gap Discovery (ABGD), the generalized mixed Yule-coalescent (GMYC) model, and the Bayesian implementation of the Poisson Tree Processes (bPTP) model, we found that species estimates ranged from 28 to 58. Among these methods, ASAP proved to be the most effective for our dataset, identifying 58 putative species. These results reinforce our assumption that the current understanding of Chironomidae species diversity is incomplete.

## Simple Summary

Non-biting midges are tiny flies that play a key role in freshwater ecosystems, but their true diversity is not yet fully understood. Scientists estimate there could be anywhere from 6000 to 15,000 species worldwide. To explore this, we studied non-biting midge larvae in Lithuania by analyzing their DNA, a 658 bp fragment of the cytochrome c oxidae subunit I (COI) gene, which is commonly used to identify species. We collected 11, 296 individuals and successfully sequenced 109 of them from six different streams and used several methods to estimate the number of species. The results ranged from 28 to 58 species, depending on the method used. This study reveals that many species of non-biting midges remain undescribed, and our understanding of their diversity is far from complete. These findings are important because non-biting midges play a vital role in freshwater food chains, and knowing more about them can help us better protect these ecosystems.

## 1. Introduction

Non-biting midges (Chironomidae) are among the most ecomorphologically diverse and species-rich insect groups, with a global presence across almost all habitats: inland waters, marine, terrestrial environments, and every continent [1,2,3]. Due to their biology and unique adaptations, Chironomidae are equipped to thrive in extreme environments, from glacial streams or hot springs, deep caves, high mountain ranges, and freshwater bodies with minimal organic matter or heavily polluted water [4,5,6,7]. Many foundational studies emphasize Chironomidae’s value as a bioindicator for assessing the ecological health of aquatic systems [8]. Among aquatic macroinvertebrates, Chironomidae is often the most abundant in both individual numbers and species diversity [9,10,11]. With over 6000 species inhabiting a broad array of habitats and ecological niches, they exhibit extensive trophic adaptations and lifestyles [12]. Notable differences in length–mass relationship models for the same Chironomidae species across various geographic regions—potentially attributable to environmental and trophic conditions, genetic variation [13,14,15,16], or methodological disparities [17]—underscore the need for models that are both taxon- and environment-specific.

Identifying Chironomidae larvae through morphological characteristics is complex and highly time-intensive, requiring expert taxonomists with specialized skills [18,19,20,21]. However, the decline in taxonomic expertise and limited funding for large-scale manual identification have made this approach increasingly challenging and time-consuming [22,23,24]. Additionally, long-term studies have reported misidentifications [25,26,27,28,29]. As a result, molecular methods, particularly DNA barcoding, are gaining importance as identification tools with studies showing an 80–90% alignment with traditional taxonomy, proving it to be a valuable supplement [30,31,32,33,34,35]. However, for molecular methods to be reliable, their results must closely match those of morphological identifications [36]. Challenges persist, including gaps in DNA sequence databases and concerns about sequence accuracy. Recent research suggests that while both morphological and molecular methods have limitations, combining them can significantly reduce identification errors by offsetting each other’s weaknesses [36,37,38].

The mitochondrial genome (mitogenome) is one of the most widely used methods for studying Diptera phylogeny, evolution, and molecular species identification, including in the family Chironomidae. However, comprehensive studies on the mitogenome of Chironomidae remain limited, and it is still unclear whether the mitogenome can efficiently resolve phylogenetic relationships at the subfamily level [39]. The COI gene is one of the most widely used mitogenome markers for species identification and phylogenetic analysis of Chironomidae and is applied to the same or closely related genera as in our study [19,40,41].

To date, approximately 190 Chironomidae species have been recorded in Lithuania [42,43,44,45]. However, most studies have focused on isolated identifications [44], and comprehensive investigations into this family remain scarce. Only recently has there been a focus on Chironomidae diversity in Lithuania [45]. This study aims to improve our understanding of the diversity, distribution, and evolutionary relationships of Chironomidae species in this region.

## 2. Materials and Methods

### 2.1. Sampling and Identification

Our study was conducted across six streams—Dubinga, Kiauna, Luknelė, Plaštaka, Skerdyksna, and Šešuola—located within the Žeimena and Šesuola sub-basins in Lithuania during 2021 and 2022, from May to September. Samples of larvae were collected from 24 locations, with four sites in each stream, ranging from sections from upstream to downstream. Streams lengths ranged from 13.6 km to 18.1 km. Larvae of non-biting midges were sampled biweekly using a D-shaped aquatic net with 1 mm mesh. Sampling was performed in randomly selected 1 m^2^ areas using the Kick Sampling method [46]. The collected samples were stored in 2 L zip-lock bags filled with 99% propylene glycol and kept at 4 °C at the Life Sciences Centre, Vilnius University.

Specimens identified as Chironomidae were sorted and preserved in 97% ethanol. Identification of Chironomidae larvae was conducted using morphological taxonomic keys [47,48,49], with systematics and taxonomy aligned to [49].

### 2.2. DNA Extraction, PCR and Sequencing

For molecular analysis, 200 samples were randomly selected, ensuring that each morpho group was represented by at least one individual, provided no additional specimens were available for that morpho group. Of these, 109 samples were successfully sequenced. Total genomic DNA was extracted from each larva using the DNeasy Blood and Tissue Kit (Qiagen, Shenzhen, China) according to the manufacturer’s instructions. Partial sequences of the 658 bp of mitochondrial cytochrome c oxidase subunit I (COI) gene were amplified using the primers LCO-1490 and HCO-2198 [50]. PCR amplification was performed in a thermal cycler (Eppendorf: Mastercycler nexus, Hamburg, Germany) with 30 μL reaction volumes containing 3 μL of genomic DNA, 1.5 μL of each primer (0.5 μM), 15 μL of DreamTaq PCR Master Mix (Thermo Scientific, Waltham, MA, USA), and 9 μL of nuclease-free water (Thermo Scientific). The thermal cycling protocol included an initial denaturation at 95 °C for 3 min, followed by 35 cycles of denaturation at 95 °C for 30 s, annealing at 49 °C for 30 s, and extension at 72 °C for 60 s, with a final extension at 72 °C for 10 min.

PCR products were purified using the GeneJet PCR purification kit (Thermo Scientific) and sequenced at Macrogen Europe BV (Amsterdam, The Netherlands). The same primers used for amplification were also used for sequencing. DNA sequences for each specimen were aligned with the BioEdit Sequence Alignment Editor [51] and compared using BLASTN [52]. All sequences were submitted to GenBank (Accession numbers: PQ458064–PQ458172).

### 2.3. Genetic Distances and Phylogenetic Analysis

Sequences were grouped according to both morphological and molecular identification to assess within-species and between-species genetic distances, calculated as the proportion of differences (p-distances) in MEGA-X [53]. For the initial data analysis, tests were carried out with and without an outgroup (*Culicoides arakawae*, Gen bank Accession number: MH135788.1). The results showed that the outgroup does not affect the topology of the phylogenetic trees. Therefore, in the study’s final results, the trees used were without the outgroup. For the phylogenetic analysis, the GTR + G + I substitution model was chosen in MEGA-X [53]. An ultrametric tree was constructed by estimating a maximum clade credibility tree using an uncorrelated lognormal relaxed clock with a mean 1.0 and standard deviation of 0.33, birth–death tree prior, implemented in BEAST v1.10.4 [54].

Two Markov chain Monte Carlo (MCMC) chains were run for 100 million generations, sampling every 1000 generations. The resulting posterior trees were combined using LogCombiner v1.10.4 [54] after discarding a 10% burn-in. Convergence diagnostics were assessed in Tracer v1.7.2 [54]. The combined posterior trees were summarized with common ancestor node heights using TreeAnnotator 1.10.4 [54]. The final tree was visualized using the baltic v.0.2.2 library in Python3 [55].

### 2.4. Species Delimitation Methods

For DNA sequence-based species delimitation, we used four methods: Assemble Species by Automatic Partitioning (ASAP) [56], Automatic Barcode Gap Discovery (ABGD) [57], the generalized mixed Yule-coalescent (GMYC) model [58], and the Bayesian implementation of the Poisson Tree Processes (bPTP) model [59].

ASAP [56] is a distance-based method that does not require prior biological insight into intraspecific diversity. We analyzed partial COI sequences of Chironomidae samples using the graphic web version of ASAP [60] with the K2P model to calculate distances (last accessed: 21 August 2024).

ABGD [57] is also a distance-based method, relying on default minimum and maximum genetic distances or those defined by the user. We used the web version of ABGD (https://bioinfo.mnhn.fr/abi/public/abgd/abgdweb.html (accessed on 22 May 2023)) with the K2P model to calculate distance parameters set by default.

The GMYC model [58] is a tree-based method applied to an ultrametric maximum clade credibility (MCC) tree constructed as described previously. The GMYC model was implemented via the R v. 4.4.0. package SPLITS (https://r-forge.r-project.org/projects/splits/ (accessed on 15 January 2009)), resulting in species-level groups.

The bPTP model [59] is another tree-based method that uses a tree in Newick format (as previously described). We analyzed the tree on the bPTP web server [61], with all parameters set to default except for the number of generations, which was adjusted to 300,000.

## 3. Results

### 3.1. Within-Species Genetic Divergences

A total of 11,296 individuals were collected from 24 sites across six streams. DNA was extracted from 200 Chironomidae specimens, resulting in successful COI fragment sequencing for 109 specimens. The 109 individuals used for molecular analysis were divided into 56 morpho groups and, following the analysis of DNA sequences, were classified into 56 species from 33 genera within four subfamilies, with 29 species represented by a single individual. For the remaining 27 species, intraspecific (within-species) genetic distances, expressed as base differences per site (p-distances), were calculated for samples from the Žeimena and Šešuola sub-basins (Table 1). The mean pairwise divergence between specimens of congeneric species was 0.81%, ranging from 0.00% to 9.94%, with 11.11% of the 18 species pair comparisons showing genetic divergences greater than 2%.

For the seven genera in which two or more species were found, interspecific (between-species) genetic distances, expressed as base differences per site (p-distances), were calculated for the samples from the Žeimena and Šešuolė sub-basins (Table 2). Most of the species showed clear genetic differentiation, with the exceptions of Chironomus cingulatus Meigen, 1830-Chironomus piger Strenzke, 1959 and Procladius culiciformis (Linnaeus, 1767)-Procladius pectinatus (Kieffer, 1909), which were not distinctly distinguished.

### 3.2. Maximum Credibility Clade Tree

**Figure 1:**
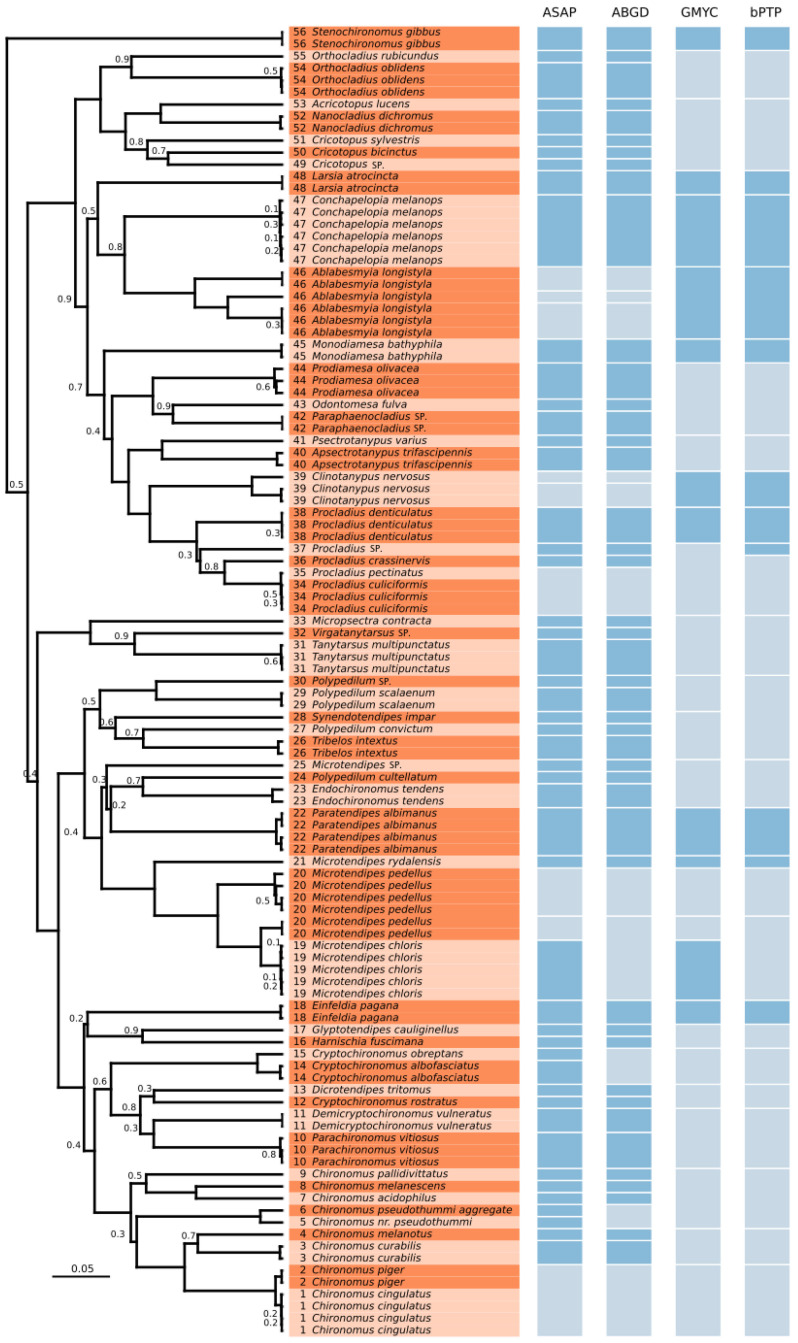
Maximum Credibility Clade tree of the Chironomidae morpho and COI sequences (**left**) and the results of species delimitation methods (**right**). Posterior probability values at nodes are shown if <0.95. The two colours highlighting the species names on the tree are used to visually separate consecutive morphos, and the numbers indicate the morpho groups. OTUs found using the different delimitation methods are separated by a white horizontal border; dark blue filling indicates the OTU agrees with the morpho and GenBank identification shown in the tree, whereas light blue filling indicates the OTU does not agree.

Among genera represented by multiple species, there is a tendency for species within each genus to cluster together on the phylogenetic tree (Figure 1). Specifically, species from the genera *Orthocladius*, *Cricotopus*, *Procladius*, and *Chironomus* each formed distinct clusters. A similar clustering pattern was observed within the genera *Microtendipes* (with the exception of samples identified as *Microtendipes* sp.) and *Cryptochironomus* (excluding samples of *Cryptochironomus rostratus* Kieffer, 1921). However, *Polypedilum* species did not form cohesive groups, indicating a lack of clear phylogenetic clustering among its representatives (Figure 1).

The analysis of interspecific mean genetic distances revealed two instances of exceptionally low p-distances (≤1%), where two groups could be considered as one: *Procladius culiciformis* with *Procladius pectinatus* (Distance: 0.22%), and *Chironomus cingulatus* with *Chironomus piger* (Distance: 0.76%).

The ASAP analysis identified 10 partitions, ranking them based on the lowest ASAP score. The optimal partition divided the dataset into 58 groups, achieving a score of 4.0 and a *p*-value of < 0.05. The second-best partition identified 56 groups with a score of 5.0 and a *p*-value < 0.05. A plot of the ASAP score against the threshold distance demonstrated that partitions with clustering distance values of 0.02 (dc = 0.02) corresponded to 58 groups.

Among subfamilies, Chironominae exhibited the highest diversity, with 34 groups in the best partition of 58 total groups. Tanypodinae followed with thirteen groups, Orthocladiinae with eight groups, and Prodiamesinae was the least diverse, represented by three groups.

The ABGD analysis applied the Kimura (K80) model with a transition/transversion ratio of 2.0, using parameters of Pmin = 0.001, Pmax = 0.10, 10 steps, and 20 bins for the distance distribution. This approach classified the dataset into 55 groups, with a K80 MinSlope value of 1.00, a barcode gap distance of 0.045, and an initial partition set at a prior maximal distance of *p* = 0.0599. Among these, the subfamily Chironominae exhibited the highest diversity, comprising thirty-one groups, followed by Tanypodinae with eleven groups, Orthocladiinae with eight groups, and Prodiamesinae as the least diverse, with three groups.

The single-threshold GMYC model identified 29 maximum-likelihood (ML) entities (confidence interval = 22–41), including 28 ML clusters (confidence interval: 21–34) and one singleton. While differences in topology were observed between the Bayesian inference tree and the maximum-likelihood tree, the GMYC analysis effectively grouped entities into genera and/or closely related clades, providing valuable insights into phylogenetic relationships.

The bPTP analysis identified 28 OTUs using the Bayesian approach, generating two output trees: one based on maximum-likelihood (24 OTUs) and another Bayesian tree (28 OTUs). Both trees exhibited topologies distinct from those of the MEGA X phylogenetic tree and the ultrametric tree used in the GMYC analysis. Compared to the GMYC, the bPTP method resulted in fewer splits among closely related OTUs and grouped sequences into genera or similar clusters, yielding outcomes significantly different from the phylogenetic trees well as the ASAP and ABGD results.

The number of OTUs identified by each method varied as follows. ASAP identified 58 OTUs (ASAP score: 4.0, *p*-value < 0.05), ABGD identified 55 OTUS (barcode gap distance = 0.045, initial partition *p* = 0.0599), GMYC identified 29 OTUs, and bPTP identified 28 OTUs (Figure 1; Supporting Information: [62]). A comparative analysis of these methods revealed that only eight OTUs, accounting for 14.29% of the phylogenetic tree, were consistently classified across all methods.

The higher OTU count in ASAP analysis (58) reflected greater subdivisions within the subfamily Chironominae, whereas the ABGD’s 55 OTUs showed fewer divisions within the same subfamily. The GMYC and bPTP analyses identified 29 and 28 OTUs, respectively, with identical delimitations in 24 cases. The ASAP and ABGD analyses exhibited similar delimitations in 52 OTUs. Overall, ASAP produced the highest OTU count, while bPTP reported the lowest.

## 4. Discussion

The objectives of this study were to explore interspecific variation and species delimitation within the family Chironomidae using the morphological identification and COI gene fragment, evaluate its diversity, and compare the effectiveness of molecular methods for species discrimination. Within-species analysis highlighted two potential identification discrepancies: *Ablabesmyia longistyla* and *Clinotanypus nervosus* (Meigen, 1818). Although these species were correctly identified based on morphological traits and sequence comparisons with GenBank data, it is plausible that their morphological identification remains ambiguous. This ambiguity likely arises from the presence of cryptic variation, overlapping diagnostic traits, potential misidentifications in reference databases, or discrepancies between morphological and molecular data. Species in the genus *Ablabesmyia* are known notoriously challenging to differentiate at the species level, with *A. longistyla*, *A. monilis* (Linnaeus, 1758), and *A. phatta* (Egger, 1864) often being easily confused. While morphological evidence supports the identification of *A. longistyla*, it raises questions about whether all species within the genus have been fully documented or described.

Despite most species exhibiting clear genetic differentiation, *Chironomus cingulatus* Meigen, 1830, *Chironomus piger* Strenzke, 1959, *Procladius culiciformis* (Linnaeus, 1767), and *Procladius pectinatus* (Kieffer, 1909) were not distinctly separated. This lack of differentiation may result from recent divergence, incomplete lineage sorting, or hybridization events. Additionally, mitochondrial introgression or the COI gene’s limited resolution for these taxa could contribute to the observed overlap. Such cases underscore the need to integrate multiple genetic markers and morphological traits for more robust species delimitation.

Conversely, GMYC and bPTP analyses indicated that *A. longistyla* individuals constitute a single species, aligning with traditional morphological identification concepts. *Clinotanypus nervosus*, distinguished by pronounced morphological features, is more readily identifiable at the species level. The sequences for this species were consistent with GenBank data, and GMYC and bPTP analyses also supported its classification as a single species. The deviation of one sequence from the 2% threshold likely reflects sequencing errors rather than genuine biological divergence.

Our findings demonstrate that distance-based algorithms are better suited for species delimitation in this context compared to tree-based methods. Specifically, the ABGD and ASAP analyses provided more reliable models for delineating species, whereas GMYC and bPTP tended to aggregate genera and broader taxonomic levels rather than distinguishing individual species. This outcome may be linked to the relatively small size of the study population, a condition under which the success rate of certain methods is known to improve [63,64]. Supporting our results, [65] also observed the strong performance of ABGD in analyzing the *Polypedilum* COI barcode dataset.

Distance-based methods, however, face a significant limitation: the absence of a universal genetic threshold applicable across all taxa [66]. Genetic variation within and between species varies widely across taxonomic groups, complicating the establishment of a single threshold for species delimitation. This highlights the necessity of complementing distance-based approaches with alternative methods. By integrating multiple analytical frameworks, the accuracy and reliability of species assignments can be improved, offering a more robust evaluation of species diversity and delimitation within a genus or taxonomic group. Another drawback of distance-based methods is their failure to account for evolutionary relationships in their algorithms [67].

In contrast, tree-based methods like GMYC and bPTP incorporate evolutionary relationships and branch length distributions, enhancing their utility in certain contexts [65]. These approaches are not limited by fixed genetic thresholds, allowing them to infer species boundaries in alignment with phylogenetic relationships, potentially offering deeper insights into evolutionary patterns.

One particularly contentious result of our study involved the placement of species within subfamilies. For instance, the Maximum Credibility Clade tree positioned *Stenochironomus gibbus* (Fabricius, 1794) near the subfamily Orthocladiinae, despite this species forming its own distinct branch. This placement might reflect the monophyletic nature of the genus [68]. Another noteworthy case was observed in the tribe Pentaneurini (subfamily Tanypodinae), where three species—*Larsia atrocincta* (Goetghebuer, 1942), *Conchapelopia melanops* (Meigen, 1818), and *Ablabesmyia longistyla*—were positioned between the subfamilies Orthocladiinae and Prodiamesinae. This unusual positioning may be attributed to the close phylogenetic relationship between Tanypodinae and Prodiamesinae [39]. Similarly, a member of Orthocladiinae (*Paraphaenocladius* sp.) was found between Prodiamesinae and Tanypodinae, suggesting potential phylogenetic links among these subfamilies.

Although morphological and COI gene analyses aid in species delimitation and phylogenetic studies, our data, along with findings from other studies, indicate that the family Chironomidae remains understudied. Furthermore, our research suggests that comprehensive morphological and genetic analyses using multiple genes, such as 16S, 28S rRNA, CAD, and others, are necessary to better investigate and understand the species diversity and phylogeny of Chironomidae [19,39,40,41].

These findings underscore the complexities and unresolved questions in the phylogenetic and evolution of the family Chironomidae. As demonstrated in this and similar studies [69], challenges related to species concepts and the limitations of current phylogenetic methods remain significant. To overcome these issues and improve species delimitation, future research should incorporate larger populations samples, adult morphological analyses, and additional genetic markers, such as 28S rRNA. Furthermore, integrating multilocus approaches and coalescent-based methods could provide deeper insights into evolutionary relationships and help resolve taxonomic uncertainties within the group.

## Figures and Tables

**Table 1:** Within-species genetic divergences expressed as the base differences per site (p-distances) calculated for the set of 27 species sampled in the Žeimena and Šventoji sub-basins.

Subfamilies	Species	No. of Individuals	Ave (%)	Min. (%)–Max. (%)
**Tanypodinae**	*Clinotanypus nervosus*	3	2.27	0.22–4.32
	*Apsectrotanypus trifascipennis*	2	0.86	0.86–0.86
	*Ablabesmyia longistyla*	6	7.43	0.00–9.94
	*Conchapelopia melanops*	6	0.09	0.00–0.22
	*Larsia atrocincta*	2	0.00	0.00–0.00
	*Procladius culiciformis*	3	0.00	0.00–0.00
	*Procladius denticulatus*	3	0.00	0.00–0.00
**Prodiamesinae**	*Monodiamesa bathyphila*	2	0.22	0.22–0.22
	*Prodiamesa olivacea*	3	1.30	1.30–1.30
**Orthocladiinae**	*Nanocladius dichromus*	2	0.22	0.22–0.22
	*Orthocladius oblidens*	3	0.11	0.00–0.22
	*Paraphaenocladius* sp.	2	0.00	0.00–0.00
**Chironomidae**	*Chironomus cingulatus*	4	0.00	0.00–0.00
	*Chironomus curabilis*	2	0.43	0.43–0.43
	*Chironomus piger*	2	0.22	0.22–0.22
	*Cryptochironomus albofasciatus*	2	0.00	0.00–0.00
	*Demicryptochironomus vulneratus*	2	0.00	0.00–0.00
	*Einfeldia pagana*	2	0.22	0.22–0.22
	*Endochironomus tendens*	2	1.73	1.73–1.73
	*Microtendipes chloris*	5	0.05	0.00–0.22
	*Microtendipes pedellus*	6	0.5	0.00–0.86
	*Parachironomus vitiosus*	3	0.22	0.22–0.22
	*Paratendipes albimanus*	4	0.86	0.22–1.30
	*Polypedilum scalaenum*	2	0.22	0.22–0.22
	*Stenochironomus gibbus*	2	0.00	0.00–0.00
	*Tribelos intextus*	2	0.86	0.86–0.86
	*Tanytarsus multipunctatus*	3	0.22	0.22–0.22

Average (Ave), Minimum (Min), Maximum (Max).

**Table 2:** Within-genus genetic divergences expressed as the base differences per site (p-distances) calculated for the set of 7 genera sampled in the Žeimena and Šventoji sub-basins.

Genera	No. of Species	Ave (%)	Min. (%)–Max. (%)
***Procladius***	5	7.80	0.22–11.23
***Cricotopus***	3	12.31	10.58–13.39
***Orthocladius***	2	12.81	12.74–12.96
***Chironomus***	8	13.92	0.65–17.06
***Cryptochironomus***	3	10.80	3.67–15.12
***Microtendipes***	4	11.78	2.16–14.25
***Polypedilum***	4	14.45	11.45–17.06

Average (Ave), Minimum (Min), Maximum (Max).

## Data Availability

All sequences used in this study are freely available at NCBI. The list of the accession numbers is here (SUB14782546). The code used for tree visualization is available on GitHub (https://github.com/Lau-Sta/Species-delimitation (accessed on 16 October 2024)) and as part of the Supplementary Materials.

## Supplementary Materials

The following supporting information can be downloaded at: https://doi.org/10.6084/m9.figshare.28172132.v1, accessed on 21 August 2024. Combined_trees.trees: Posterior tree distribution combined from the two MCMC chains ran for the Bayesian analysis. mcct_tree_1.nwk: Maximum clade credibility tree from the combined tree distribution. mcct_mainfig.*: PDF and SVG files for Figure 1 in the main manuscript. mcct_plotting.*: HTML and Jupyter Notebook with the Python code for producing the figures in the manuscript and Supplementary Materials. mcct_suppmatfig.*: PDF and SVG files for the figure in Supplementary Material, showing all posterior distribution values in the phylogeny. NTree*: XML files used for running the Bayesian analysis in Beast and the posterior tree distributions for each MCMC chain. Table2_2.csv: File with morphotype and OTU classification for each sequence in the MCC tree and the annotations used for plotting.
